# DEATH ANXIETY AND FINANCIAL DECISION-MAKING IN AGING: A STUDY FROM THE HUMAN CONNECTOME PROJECT AGING (HCP-A)

**DOI:** 10.1093/geroni/igz038.3311

**Published:** 2019-11-08

**Authors:** Timothy K Ly, Mirella Diaz-Santos, Liam Campbell, Marcela Caldera, Taylor Kuhn, Susan Bookheimer

**Affiliations:** University of California, Los Angeles, Los Angeles, California, United States

## Abstract

While research addressing late-life death anxiety (the fear of death or the dying process) has focused on end-of-life care decision-making, few have studied the effect of late-life death anxiety on financial decision-making. This is particularly relevant to financial decision-making as older adults are more vulnerable to fraud and deception. The aim of this study was to determine how age and death anxiety affect financial decision-making in a sample of older adults of 60-93 years of age (N = 102), who participated in the HCP-A project at UCLA. To study this relationship, we used a delayed reward discounting task to model financial decision-making, where higher rates of discounting indicate a greater preference for immediate, smaller monetary rewards and lower rates of discounting indicate more future-oriented planning. To account for age-related cognitive decline, cognitive functioning was assessed using the NIH Toolbox. We hypothesized that the presence of death anxiety will increase discounting of future rewards in older adults. Results from a univariate ANOVA showed an interaction between age, death anxiety, and delayed reward discounting. Specifically, older adults with self-reported death anxiety showed greater preference for immediate, smaller monetary rewards. By controlling for cognition, these findings suggest that death anxiety moderates decision-making in late-life adults and may add to our understanding of why older adults are more susceptible to financial abuse. These results suggest a need to consider death anxiety as a moderating variable when developing and implementing policies and services that are geared towards older adults.

